# Comparative LC-MS/MS-based profiling of phytohormones: a unified analytical approach across diverse plant matrices

**DOI:** 10.3389/fpls.2025.1670979

**Published:** 2025-09-19

**Authors:** Muhammad K. Hakeem, Tamilarasan Rajendaran, Esam Eldin Saeed, Ajay K. Mishra, Khaled M. Hazzouri, Iltaf Shah, Khaled M. A. Amiri

**Affiliations:** ^1^ Department of Chemistry, College of Science, United Arab Emirates University, Al Ain, United Arab Emirates; ^2^ Khalifa Center for Genetic Engineering and Biotechnology, United Arab Emirates University, Al Ain, United Arab Emirates; ^3^ Department of Biology, College of Science, United Arab Emirates University (UAEU), Al Ain, United Arab Emirates

**Keywords:** LCMS (liquid chromatography-mass spectrometry), phytohormones, plant matrices, sustainability, LCMS/MS analysis

## Abstract

**Introduction:**

Phytohormones are critical regulators of plant growth, development, and stress responses. Despite advancements in analytical techniques, profiling multiple phytohormones across various plant matrices using a standardized approach remains underexplored.

**Methods:**

This study presents a unified LC-MS/MS analytical platform, employing consistent chromatographic and mass spectrometric conditions in combination with tailored matrix-specific extraction procedures, to profile and quantify key phytohormones. This includes abscisic acid (ABA), salicylic acid (SA), gibberellic acid (GA), and indole-3-acetic acid (IAA), across five plant matrices of significant agricultural and medicinal value. The method was validated for sensitivity, reproducibility, and matrix adaptability, demonstrating robust performance in profiling phytohormones from these diverse species.

**Results:**

The results revealed distinct phytohormonal profiles, reflecting species-specific physiological adaptations to environmental conditions. For instance, cardamom exhibited high levels of SA and ABA, associated with stress responses in arid climates, while aloe vera showed lower phytohormone levels, indicative of its drought tolerance. Statistical analyses confirmed significant variation in hormone concentrations across the matrices, emphasizing the role of both genetic and environmental factors.

**Discussion:**

This unified LC-MS/MS platform offers a comprehensive approach to understanding phytohormone distribution and dynamics, with implications for improving agricultural practices, crop resilience, and the development of functional foods and nutraceuticals. The findings contribute to the broader understanding of plant physiology and offer practical applications in sustainable agriculture, particularly in regions with significant agricultural and medicinal crop production.

## Introduction

Phytohormones, a diverse group of small organic signaling molecules, are pivotal in regulating fundamental physiological processes including plant growth, development, and stress adaptation (Salvi et al., 2021; Zhao et al., 2021; El Sabagh et al., 2022; Pal et al., 2023; Vaishnav et al., 2023). Their diverse chemical nature and dynamic regulatory functions enable plants to adapt to various environmental stresses, including drought, flooding, salinity, and pathogen infection. In agriculture, the strategic manipulation of phytohormonal pathways has profoundly enhanced crop resilience and productivity, addressing critical global challenges such as food security, sustainability, and climate change adaptation (Ali et al., 2024; Kaya, 2024; Sanaullah, 2024; Shaukat et al., 2024). For instance, modulation of abscisic acid (ABA) and gibberellin (GA) signaling pathways has successfully improved stress resistance and yield stability in major crops, including rice, maize, and wheat, particularly under extreme climatic conditions (Castro-Camba et al., 2022; Kishor et al., 2022; Parwez et al., 2022; Singh and Roychoudhury, 2023; Lamlom et al., 2025). Given the accelerating impacts of climate variability, hormonal profiling and modulation are vital for climate-smart agricultural practices to ensure stable food production and ecological resilience (Hirayama and Mochida, 2022; Schmidt et al., 2024; Raza et al., 2025).

Phytohormones exert a significant influence on the biosynthesis and accumulation of nutritionally and pharmaceutically valuable secondary metabolites in medicinal and edible plants. Compounds such as flavonoids, terpenoids, and phenolic acids, regulated by hormonal signals, contribute substantially to the therapeutic efficacy and nutritional quality of diverse plant species (Mohammadi-Cheraghabadi et al., 2023; Hilal et al., 2024). *Aloe vera* (aloe vera), renowned for its medicinal properties, exemplifies this relationship, as its hormonal composition directly influences its production of therapeutic metabolites. Likewise, economically important crops and medicinal herbs, including *Solanum lycopersicum* (tomato), *Plectranthus amboinicus* (Mexican mint), *Phoenix dactylifera* (dates), and *Elettaria cardamomum* (cardamom), feature distinct hormonal profiles that potentially modulate their nutritional and therapeutic qualities. Understanding these complex hormonal profiles thus holds considerable promise, not only for clarifying their biological roles but also for leveraging their applications in agriculture, nutraceuticals, and pharmaceuticals (Mutha et al., 2021; Asif et al., 2022; Sun and Shahrajabian, 2023; Sonawdekar et al., 2024).

Advances in analytical methodologies, particularly liquid chromatography-tandem mass spectrometry (LC-MS/MS), have significantly improved, emerging as a robust and precise analytical approach that provides sensitive and reliable detection of phytohormones, even at minute concentrations. Recent advances have streamlined simultaneous quantification methods for multiple phytohormones from complex matrices, significantly improving throughput and reproducibility (Hashiguchi et al., 2021; Yao et al., 2022; Cao and He, 2023; Chen et al., 2024). Despite these methodological advances, comprehensive comparative profiling across diverse plant species using a single, standardized LC-MS/MS analytical platform remains notably underexplored. Previous studies have largely focused on individual species or single hormone classes, limiting simultaneous comparative profiling and comprehensive insights into hormonal diversity across different matrices (Yang et al., 2022; Vrobel et al., 2024; Wang et al., 2024; Zhang et al., 2024). Furthermore, existing phytohormonal profiling methodologies often face challenges related to sensitivity, matrix interferences, and lengthy or complicated sample preparation procedures (Bisht et al., 2021; Kaya, 2024; Vrobel et al., 2024). Overcoming these barriers through robust analytical frameworks capable of consistent performance across diverse matrices is vital, particularly given the varied biochemical compositions of different plant species.

To bridge this critical knowledge and methodological gap, our study adopts an integrated and standardized LC-MS/MS-based analytical approach to profile multiple key phytohormones abscisic acid (ABA), salicylic acid (SA), gibberellic acid (GA), indole-3-acetic acid (IAA), and related compounds across five economically and medicinally significant plant matrices: cardamom, dates, tomato, Mexican mint, and dates. These species were deliberately selected based on their distinct agricultural, economic, and therapeutic importance in various global regions. For instance, dates and aloe vera hold particular agricultural and medicinal significance in arid climates such as those of the UAE, whereas tomato and Mexican mint are widely cultivated globally for both culinary and medicinal purposes. Cardamom, a high-value spice, also makes a significant contribution to the economy and is renowned for its therapeutic properties (Collins et al., 2022; Golmohammadi, 2022; Gupta et al., 2023; Hakeem et al., 2024).

By applying our integrated LC-MS/MS platform with standardized analytical conditions and tailored, matrix-specific extraction procedures, we aim to validate the robustness and versatility of our analytical methodology. We aimed to generate detailed hormonal profiles that illuminate the distribution and dynamics of phytohormones across species. Understanding these phytohormonal profiles will enhance our fundamental understanding of plant physiological diversity and inform agricultural practices, stress management strategies, and the development of nutraceuticals. Given the economic and medicinal importance of these plant species, particularly in agricultural and economically critical regions such as the UAE and Mediterranean countries, where these species significantly contribute to both local economies and food security, our findings have substantial implications for sustainable agricultural development and functional food enhancement. This study represents a significant advancement in plant hormonal biology, analytical chemistry, and agricultural science, promising direct implications for sustainable agriculture, crop improvement strategies, functional food innovations, and the targeted use of medicinal plants. Considering the extensive global dependence on these crops and medicinal herbs for nutritional, economic, and health purposes, this study has the potential to significantly improve agricultural policies, climate-adaptive strategies, and innovations in nutraceutical and medicinal plant research.

## Materials and methods

### Chemicals and reagents

LC-MS grade Methanol, Formic acid, and Acetic acid (LR grade) were obtained from Supelco (Germany) and Fluka (Switzerland). Milli-Q-Water obtained from In-house (UAE University). Indole-3-acetic acid, Isopentenyl adenine, Naphthalene acetic acid, indole-3-butyric acid, 6-Benzyl aminopurine, Gibberellic acid, Salicylic acid, Abscisic acid, and salicylic acid D4 (internal standard) were obtained from Sigma-Aldrich (USA).

The experiment was conducted using a SHIMADZU LC-30AD Nexera X2 system coupled with an LC-MS 8060 mass spectrometer (Shimadzu, Japan), which provided high sensitivity and precision for the analysis. A ZORBAX Eclipse Plus C18 column (4.6 x 100 mm, 3.5 μm particle size) from Agilent.

### Sample preparation and extraction

Sample preparation and extraction were optimized for each plant matrix to ensure maximal recovery of phytohormones while maintaining cross-matrix consistency suitable for LC-MS/MS analysis. Samples were homogenized with mortar and pestle under liquid nitrogen to ensure their integrity. For each matrix, approximately 1.0 g ± 0.1 g of plant material was weighed and subjected to matrix-specific extraction protocols. Detailed extraction protocols for each matrix are provided in **Supplementary Information** (**Supplementary Table S1**). Briefly, samples were homogenized and extracted with solvent mixtures tailored to each matrix, followed by centrifugation and phase separation with the addition of an internal standard (salicylic acid D4, selected for its broad ionization stability and suitability across diverse classes of phytohormones analyzed in this study). Although isotope-labeled standards specific to each compound can further improve analyte-specific correction, our approach ensured adequate normalization across matrices and was selected as a practical strategy to maintain consistency and comparability in quantification. This choice represents a balance between analytical robustness and resource feasibility, and potential incorporation of multiple isotope-labeled standards can further strengthen future studies. The dates matrix, due to its high sugar and polysaccharide content, required a two-step procedure, involving acetic acid followed by 2% HCl in ethanol. After adding extraction solvents, samples were centrifuged at 3000 × g for 10 minutes at 4°C, and the supernatant was filtered through a 0.22 µm syringe filter to remove particulate matter. The resulting extract was then diluted with mobile phase to ensure compatibility with LC-MS/MS analysis. Each matrix was treated separately to accommodate its unique chemical composition, ensuring that the phytohormones were efficiently extracted for accurate quantification. The samples were then injected into the LC-MS/MS system for detailed profiling of phytohormones, ensuring consistent and reliable data across all matrices.

### LC-MS/MS analysis

A unified liquid chromatography tandem mass spectrometry (LC-MS/MS) method was employed for the simultaneous quantification of multiple phytohormones across all plant matrices. The analysis was performed using a SHIMADZU Nexera X2 LC-30AD binary pump system coupled to a Shimadzu LCMS-8060 triple quadrupole mass spectrometer. Chromatographic separation was carried out on a ZORBAX Eclipse Plus C18 column (4.6 × 100 mm, 3.5 µm; Agilent Technologies) maintained at 30°C. The mobile phase consisted of 0.01% formic acid in water (A) and methanol (B), delivered isocratically at a ratio of 35:65 (v/v) with a constant flow rate of 0.5 mL/min. The injection volume was 10 μL, and the autosampler temperature was maintained at 5°C. The total runtime per sample was 7 minutes.

Mass spectrometric detection was performed using electrospray ionization (ESI) in both positive and negative modes, depending on the characteristics of the compound. Phytohormones were monitored using multiple reaction monitoring (MRM) mode, with compound-specific transitions selected based on signal intensity and selectivity. The optimized mass spectrometric conditions included precursor-to-product ion transitions (Q1 → Q3), a dwell time of 100 ms, Q1 and Q3 pre-bias voltages, and compound-specific collision energies. Method tuning was conducted with standard solutions (100 ng/mL) infused in a 1:1 methanol-water mixture to achieve maximum ion yield and stability for each target analyte. The method focused on major phytohormones, including indole-3-acetic acid (IAA), gibberellic acid (GA), abscisic acid (ABA), salicylic acid (SA), and the internal standard salicylic acid-D4 (SA-D4), as well as other compounds. These MRM transitions were uniformly applied across all matrices to ensure consistency and comparability. Detailed ion transition parameters and MS settings are provided in **Supplementary Table S2**. By employing a consistent LC-MS/MS workflow across chemically diverse plant tissues, this platform enabled robust, sensitive, and reproducible quantification of phytohormones in aloe vera, tomato, Mexican mint, dates, and cardamom, highlighting the method’s adaptability and analytical strength.

### Method validation

The developed LC-MS/MS method was validated in accordance with US FDA bioanalytical method validation guidelines (Research, 2020) to ensure its reliability, reproducibility, and suitability for quantitative analysis of phytohormones in diverse plant matrices. Validation parameters included linearity, limit of detection (LOD), limit of quantification (LOQ), accuracy, precision, and recovery. Three quality control levels, low, medium, and high, were used to assess intra-day and inter-day performance. Linearity was established for each analyte using matrix-matched calibration curves with seven concentration points. LOD and LOQ were calculated following US FDA bioanalytical guidelines, based on the standard deviation of the response and the slope of the calibration curve, corresponding to a signal-to-noise (S/N) ratio of 3:1 for LOD and 10:1 for LOQ. Accuracy was evaluated by calculating recovery percentages, while precision was determined by the relative standard deviation (%CV) of replicate measurements. Intra-day and inter-day accuracy across all QC levels ranged between 80% and 120%, and %CV values remained below 15%, confirming the method’s robustness. No significant matrix effects or interferences were observed, and analyte identification was confirmed by consistent retention times and multiple ion transition ratios. The method showed strong performance characteristics across multiple plant types without requiring matrix-specific re-optimization, supporting its applicability for routine analysis and comparative studies.

## Results and discussion

### Method validation results

The LC-MS/MS method for phytohormone analysis was thoroughly validated across a range of plant matrices, including cardamom, dates, tomato, Mexican mint, and aloe vera. Samples originating from different geographical regions were sourced from local markets in Al Ain, United Arab Emirates. Key analytical parameters, linearity, sensitivity, precision, accuracy, and matrix effects were assessed to ensure reliability and broad applicability. Calibration curves constructed for each phytohormone demonstrated excellent linearity over a wide concentration range, with correlation coefficients (R^2^) consistently exceeding 0.99. This strong linear relationship confirms that the method provides accurate quantification across the expected concentration spectrum in all tested matrices. Sensitivity was evaluated by determining the limits of detection (LOD) and limits of quantification (LOQ), which were found to be sufficiently low to enable the detection of target compounds at trace levels. LOD and LOQ values varied slightly across matrices (e.g., SA ranging from 1.6–8.2 ng/mL, IAA from 0.83–11.2 ng/mL). This variability is primarily attributed to differences in matrix complexity (e.g., sugar, polysaccharide, or polyphenol content), extraction efficiency, and minor variations in ionization efficiency due to matrix-induced suppression or enhancement. Instrumental factors such as batch-to-batch variability may also contribute slightly. Importantly, these differences remained within acceptable validation criteria and did not compromise the overall analytical robustness. **Table 1** summarizes key validation parameters for the analyzed phytohormones, including linearity range, correlation coefficients (R²), LOD, LOQ, precision, and accuracy. These values represent the ranges observed across all tested plant matrices, demonstrating the method’s consistent and robust analytical performance.

**Table 1:** Summary of method validation parameters for phytohormones analysis across different plant matrices.

Phytohormone	Linearity range (ng/mL)	R² range	LOD range (ng/mL)	LOQ range (ng/mL)	Precision (CV%)	Accuracy (%)
Indole-3-Acetic Acid (IAA)	1– 225	0.98 – 0.99	0.2 – 3.7	0.83 – 11.2	<10	85 – 115
Gibberellic Acid (GA)	1 – 125	0.98 – 0.99	0.3 – 4.0	1.0 – 12.2	<10	85 – 115
Salicylic Acid (SA)	1 – 500	0.98 – 0.99	0.5 – 2.7	1.6 – 8.2	<10	80 – 120
Abscisic Acid (ABA)	1 – 500	0.98 – 0.99	0.4 – 3.7	1.2 – 11.2	<10	85 – 115
6-Benzylaminopurine (6BAP)	1 – 100	0.98 – 0.99	0.5 – 4.0	1.5 – 12.1	<18	83 – 116
Indole-3-Butyric Acid (I3BA)	5 – 100	0.98 – 0.99	2.17	6.59	<13	86 – 111
Naphthalene Acetic Acid (NAA)	5 – 2500	0.98 – 0.99	2.47 – 36	7.5 – 110	<10	95 – 105
Isopentyl Adenine (IPA)	0.2 – 35	0.98 – 0.99	0.01-3.057	0.04 – 9.2	<7	89 – 99

Ranges reflect variation observed across the different plant matrices analyzed.

The method exhibited high precision, with both intra-day and inter-day coefficients of variation remaining below 10% across all analytes and matrices. Recovery experiments further confirmed the accuracy of the method, with observed values falling between 80% and 120%, which is within the accepted bioanalytical validation criteria. Potential matrix effects, i.e., signal suppression or enhancement caused by co-eluting substances, were minimal, generally within ±20%, underscoring the robustness of sample preparation and chromatographic conditions. Where minor matrix interferences were detected, matrix-matched calibration curves were employed to correct for any bias, ensuring reliable quantification. These validation results underscore the method’s high sensitivity, accuracy, and precision across multiple phytohormones and diverse botanical matrices. The ability of a single method to perform well across multiple plant types without requiring significant re-optimization underscores its suitability for comparative phytohormone studies and its potential for routine applications in plant metabolite analysis. Detailed validation data for individual matrices, including specific numerical parameters, are provided in the **Supplementary Information** (**Supplementary Tables S3**-**S7**), allowing assessment of matrix-specific performance in greater details.

### Sample analysis

**Figure 1:**
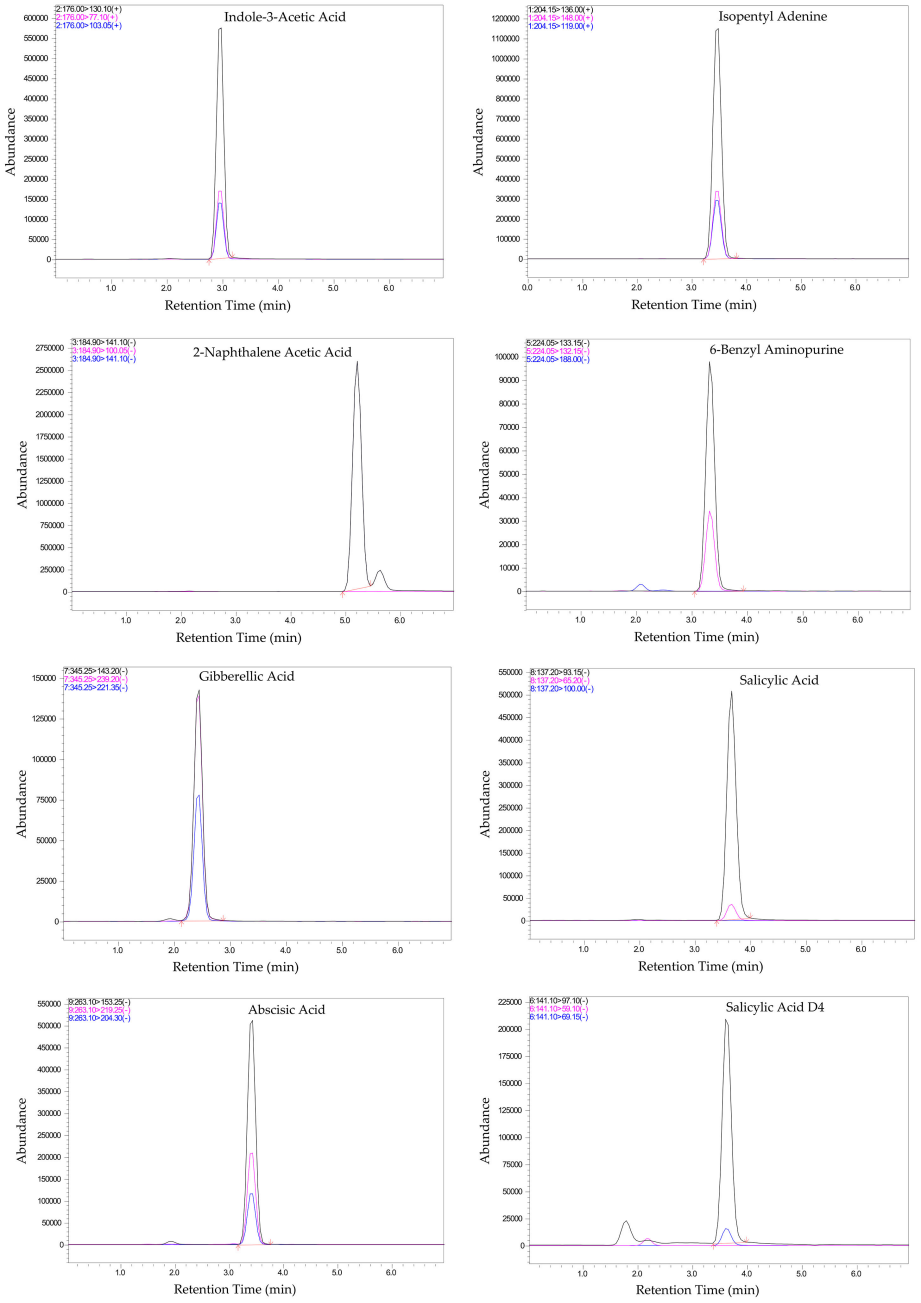
Chromatograms of Phytohormones and Internal Standards showing the separation of phytohormones with distinct retention times. Black represents the precursor ion (Q1), and blue and pink represent the product ions (Q3).

The validated LC-MS/MS method was then applied to analyze phytohormone profiles in the selected plant matrices. Representative chromatograms for each phytohormone (**Figure 1**) illustrate well-resolved peaks for all target phytohormones and internal standards, demonstrating the method’s high selectivity and sensitivity. Each phytohormone exhibited chromatographic separation at its distinct retention times, enabling explicit identification and accurate quantification in complex plant matrices. The retention times were consistent across replicate injections, demonstrating excellent reproducibility. This chromatographic profile serves as a foundation for confident detection and quantification of phytohormones in complex plant matrices, as it confirms the method’s ability to separate structurally similar compounds with diverse chemical properties effectively. Furthermore, the use of stable isotope-labeled internal standards facilitates the correction of potential matrix effects and instrumental variability, thereby enhancing the reliability of quantification.

### Cardamom phytohormone profile

**Figure 2:**
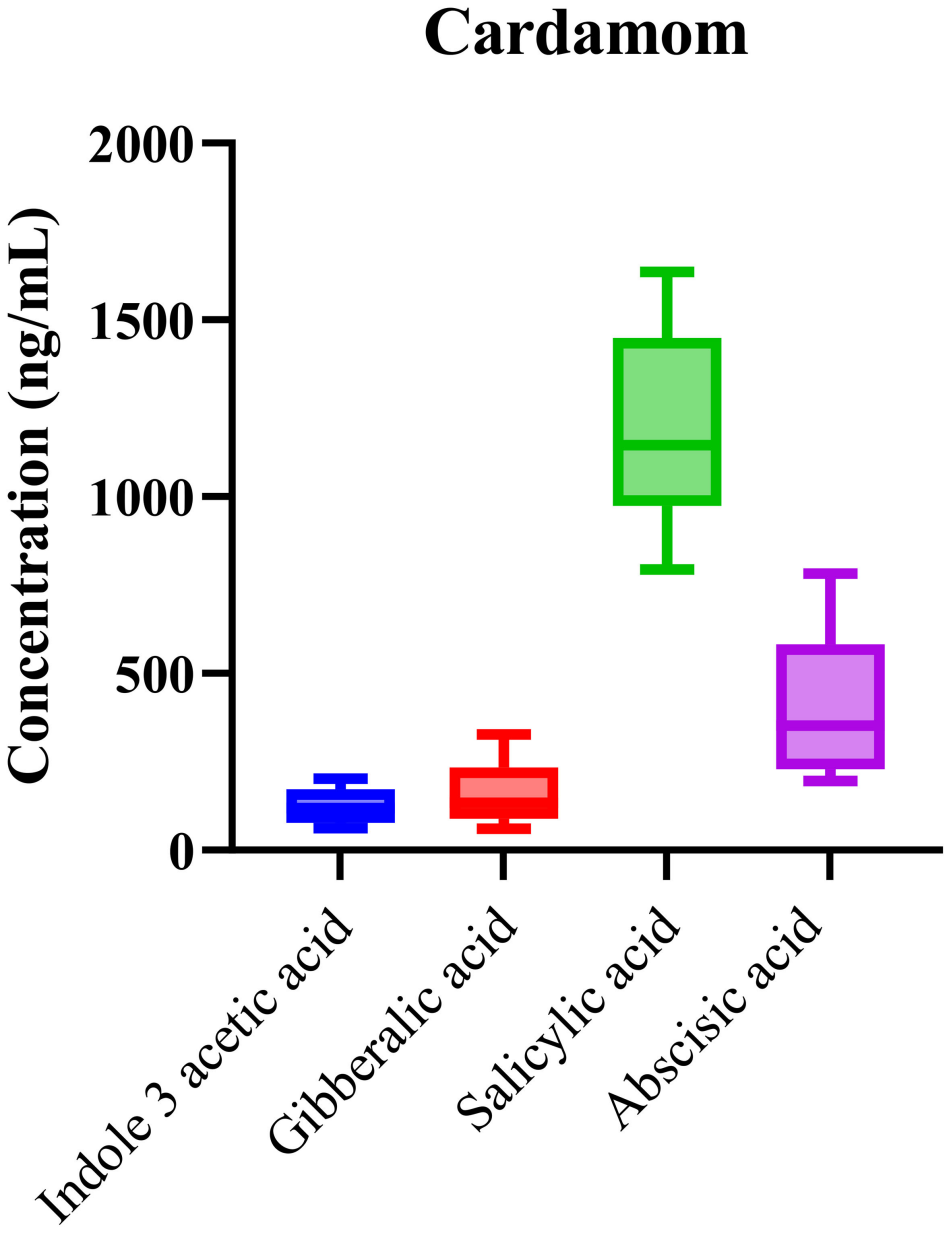
The concentrations of detected phytohormones in cardamom samples, with error bars indicating standard deviations.

The phytohormonal composition of cardamom samples, sourced from various geographic regions, was analyzed using the validated LC-MS/MS method. Consistent detection of key phytohormones, including abscisic acid (ABA), indole-3-acetic acid (IAA), gibberellic acid (GA), and salicylic acid (SA), was observed across all samples. The box-and-whisker plots (**Figure 2**) demonstrate the distribution of phytohormone concentrations, with SA showing notably higher median concentrations compared to the other analytes. This result reflects SA’s well-established role in regulating plant stress responses, seed dormancy, and developmental processes, suggesting its prominent role in cardamom’s adaptation to environmental stressors.

The high concentration of SA in cardamom suggests its prominent role in regulating stress responses, particularly in harsh growing conditions where plant defense mechanisms are activated. SA is known to regulate systemic acquired resistance (SAR), and its elevated presence may reflect the plant’s adaptation to environmental stressors. In contrast, IAA, GA, and ABA displayed more variable concentrations, suggesting that their roles in cardamom are more context-dependent, influenced by growth stages and regional cultivation practices.

Statistical analysis using one-way ANOVA (p < 0.0001) confirmed significant differences in phytohormone concentrations across cardamom samples from different geographic origins. This suggests that both environmental conditions and genetic factors contribute to the observed variability in hormone levels. The R^2^ value of 0.87 underscores the strong explanatory power of the model, reinforcing the reliability and reproducibility of the LC-MS/MS method for quantifying these hormones. The variability in phytohormonal profiles across different origins further supports that phytohormones play a vital role in plant acclimatization, particularly in response to diverse climatic and soil conditions.

### Dates phytohormone profile

**Figure 3:**
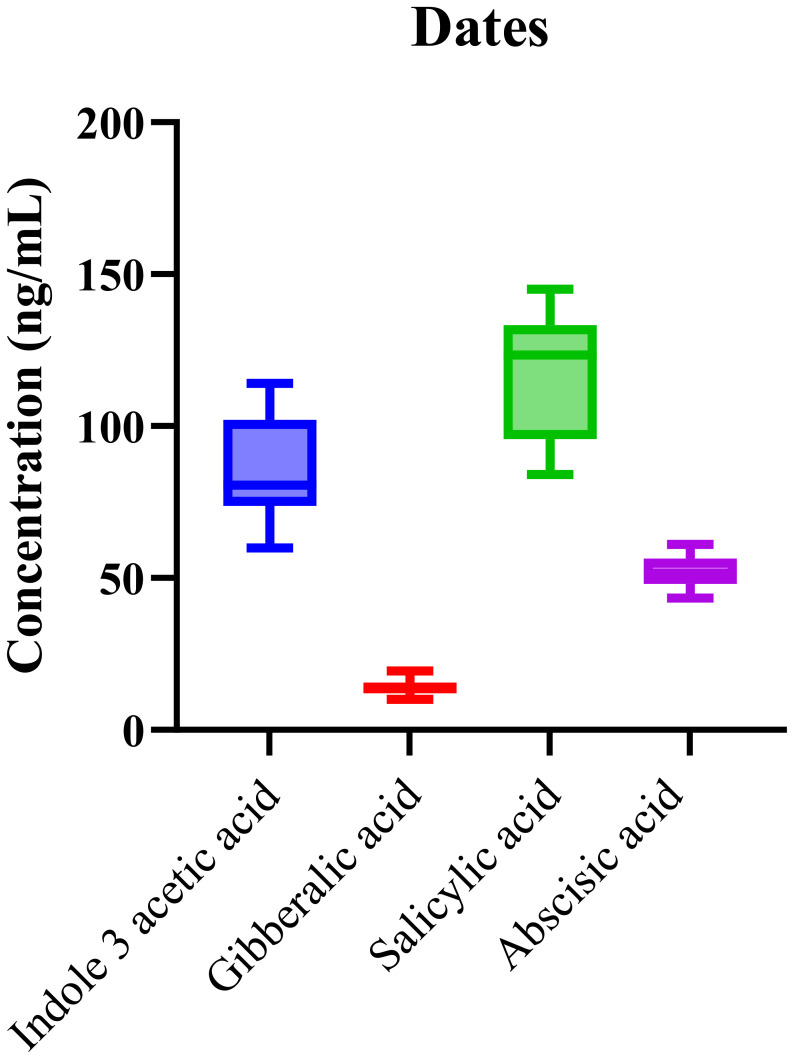
Boxplots showing the distribution of phytohormone levels in *Phoenix dactylifera* (date) samples with notably high medians for SA and IAA. Boxes represent the interquartile range (IQR), horizontal lines denote medians, and whiskers indicate data dispersion.

The validated LC-MS/MS method was also applied to analyze the phytohormone content in date samples collected from multiple regions. The analysis consistently detected IAA, GA, SA, and ABA across all samples. As shown in **Figure 3**, the box-and-whisker plot illustrates the distribution of hormone concentrations, with SA and IAA exhibiting the highest median levels. This finding aligns with the critical roles these phytohormones play in regulating fruit ripening and stress responses in date palms.

The prominent role of salicylic acid (SA) in date palms is consistent with its well-established functions in plant defense, particularly in response to abiotic stresses like heat and drought, which are common in arid environments. Elevated levels of SA may reflect the plant’s adaptation mechanisms, enhancing its resistance to stress and improving survival in harsh climatic conditions. Furthermore, SA’s involvement in systemic acquired resistance (SAR) (Zeier, 2021) suggests that its elevated presence might also be linked to the plant’s ability to withstand pathogen attacks, which is crucial for date palm cultivation in areas with varying environmental stressors. In addition to SA, indole-3-acetic acid (IAA) exhibited high concentrations, indicating its essential role in growth regulation, particularly in seedling development, cell elongation, and fruit maturation. The significant levels of IAA observed suggest that, alongside SA, it plays a role in regulating the development of date fruits, particularly during the early stages of ripening. These findings align with the known functions of IAA in promoting growth and developmental processes.

Statistical analysis using one-way ANOVA (p < 0.0001) confirmed that the observed differences in hormone concentrations were statistically significant between the geographic origins. The high R^2^ value of 0.89 demonstrates that the variations in phytohormone levels can be reliably explained by the model, further highlighting the method’s robustness in profiling these complex plant matrices. The variation observed in phytohormone concentrations, particularly between different geographic regions (p < 0.0001), emphasizes the significant influence of environmental conditions on hormone biosynthesis and accumulation. Environmental factors, such as temperature, water availability, and soil composition, likely impact the production and regulation of these phytohormones, which are crucial for regulating fruit ripening, stress adaptation, and overall plant health.

### Tomato phytohormone profile

**Figure 4:**
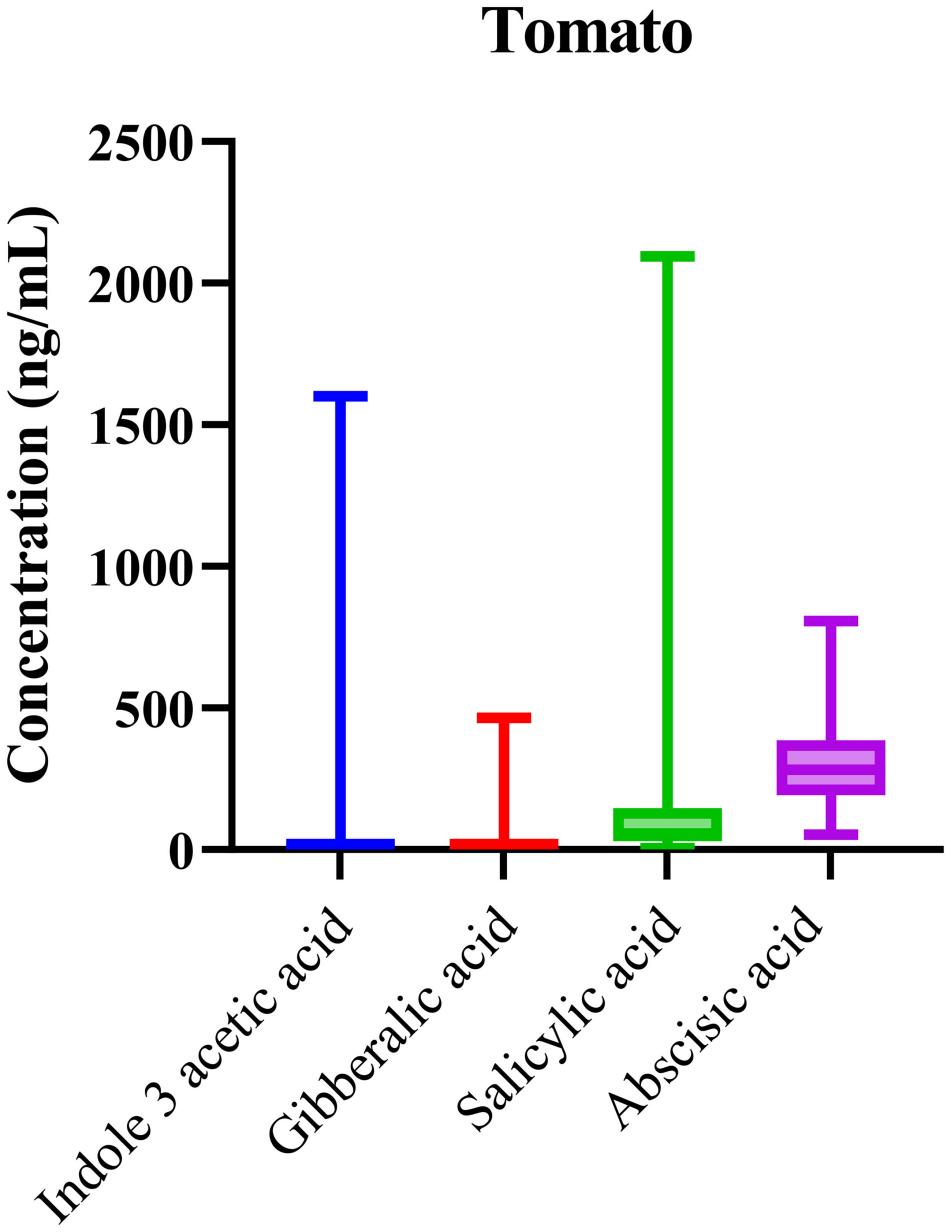
Boxplots showing variability in phytohormone profiles across *Solanum lycopersicum* (tomato) samples. Median concentrations, interquartile ranges, and overall distribution are displayed.

Phytohormonal analysis of tomato samples collected from various regions revealed the consistent presence of key phytohormones, including IAA, GA, ABA, and SA, across all samples. The box-and-whisker plots in **Figure 4** illustrate the distribution of these hormones with notable variation in their concentrations. ABA, in particular, exhibited elevated median levels, aligning with its well-established role in regulating fruit ripening and mediating stress responses in tomato plants.

The elevated levels of abscisic acid (ABA) in tomato samples strongly correlate with its known role in fruit ripening, especially under stress conditions such as water scarcity. ABA regulates various processes during fruit maturation, including seed dormancy, ripening, and response to dehydration. The prominence of ABA in these samples suggests its pivotal role in the tomato’s response to the environmental challenges of the regions where the samples were sourced. This finding aligns with existing literature, which highlights ABA’s involvement in stress responses and its role as a key regulator of fruit ripening. In tomatoes, ABA’s high concentration during ripening is necessary for maintaining fruit quality and ensuring seed maturation, particularly in conditions where water stress is a concern.

Statistical analysis confirmed the significant variability across samples (p < 0.0001), with one-way ANOVA showing that the differences in hormone concentrations were highly significant. This reinforces the idea that genetic and environmental factors play a crucial role in shaping the hormonal landscape in tomatoes. The variability observed suggests that tomatoes from different regions experience distinct physiological processes due to the differences in climate, water availability, and soil quality, further underlining the complex interactions between genetics and environment in shaping phytohormonal regulation.

### Mexican mint phytohormone profile

**Figure 5:**
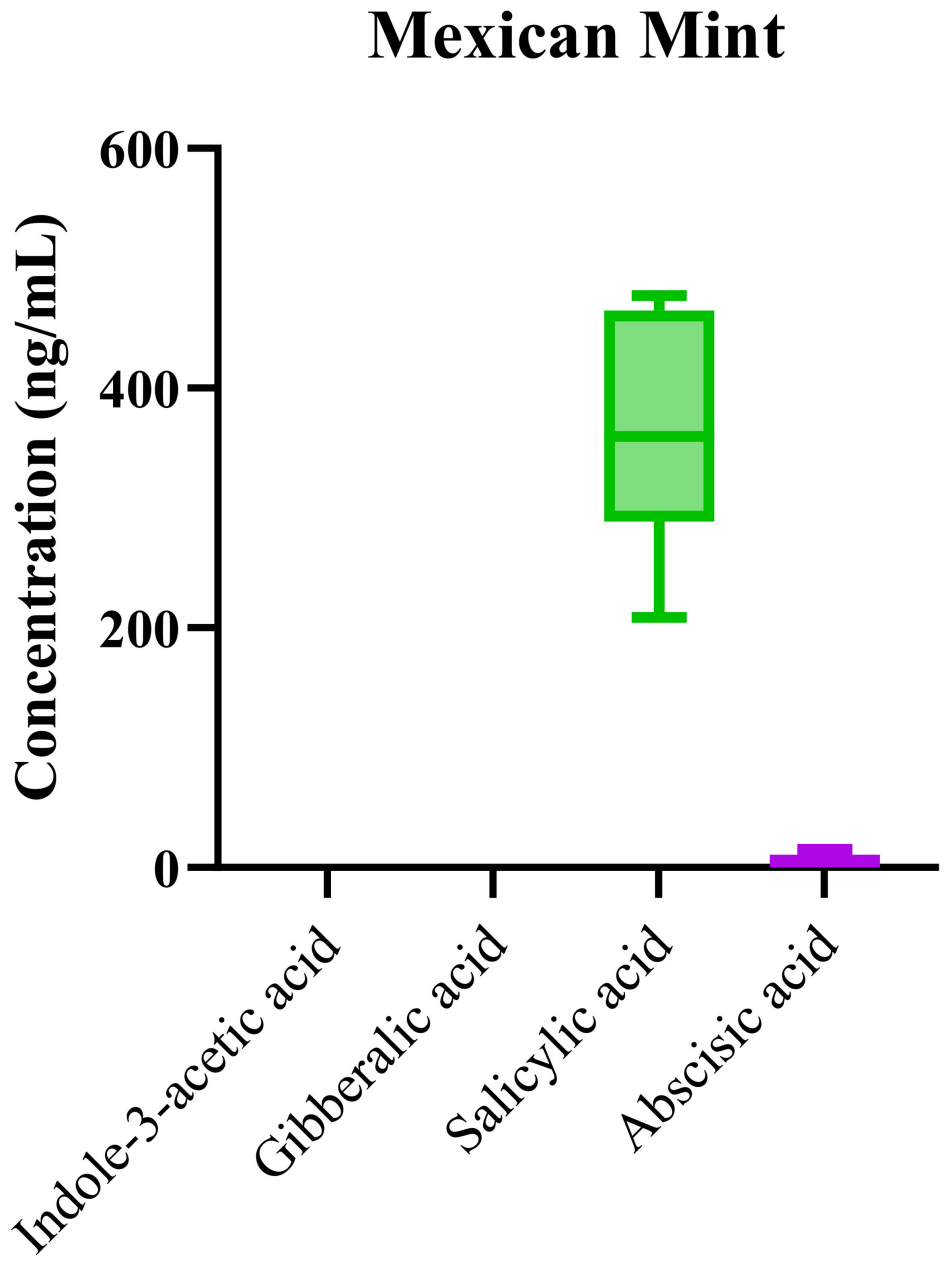
Box-and-whisker plots of phytohormone concentrations in *Plectranthus amboinicus* (Mexican mint) samples.

The LC-MS/MS method was further employed to profile phytohormones in Mexican mint samples. Box-and-whisker plots in **Figure 5** illustrate the distribution of hormone concentrations. Among the phytohormones, SA exhibited the highest median concentration, indicating its dominant role in the plant’s stress response. In contrast, ABA levels were very low, and IAA and GA were found to be below the limit of detection in most samples. Salicylic acid (SA) is well-known for its critical role in plant defense, particularly in response to biotic stresses such as pathogen attack. The elevated levels of SA in Mexican mint likely reflect the plant’s enhanced resistance mechanisms to environmental stressors. This is particularly relevant since Mexican mint, a medicinal plant often grown in areas with varying climatic conditions, is frequently exposed to pathogens, herbivores, and other stressors. The high concentration of SA observed in this study supports the hypothesis that it plays a central role in the plant’s stress defense and immune response. Additionally, SA’s involvement in systemic acquired resistance (SAR) suggests that its elevated concentration in these samples may enhance the plant’s capacity to withstand environmental challenges.

The low levels of ABA observed in the samples also suggest a potential trade-off in the hormonal balance. ABA is typically associated with stress responses, particularly in regulating water loss and stomatal closure during drought conditions. In contrast to SA, indole-3-acetic acid (IAA) and gibberellic acid (GA) were found at concentrations below the limit of detection. This is noteworthy, as both IAA and GA are key hormones involved in growth regulation and cell elongation, processes that are crucial during normal vegetative and reproductive development. The low concentrations of these hormones may reflect the plant’s growth strategy under stress conditions, where the plant might prioritize defense mechanisms over growth. This prioritization of defense over growth could be an adaptive response to environmental stress, as the plant shifts resources toward survival rather than expansion.

The significant differences in phytohormone levels, as revealed by the one-way ANOVA (p < 0.0001), highlight the strong influence of environmental factors such as temperature, water availability, and cultivation practices on phytohormone regulation. This finding underscores the importance of environmental context in shaping the hormonal profiles of plants, especially medicinal species like Mexican mint, where phytochemical content is directly linked to its therapeutic properties. The high R^2^ value of 0.89 further confirms that the LC-MS/MS method is both robust and sensitive, allowing for reliable quantification of even low-concentration phytohormones in complex herbal matrices like Mexican mint.

### Aloe vera phytohormone profile

**Figure 6:**
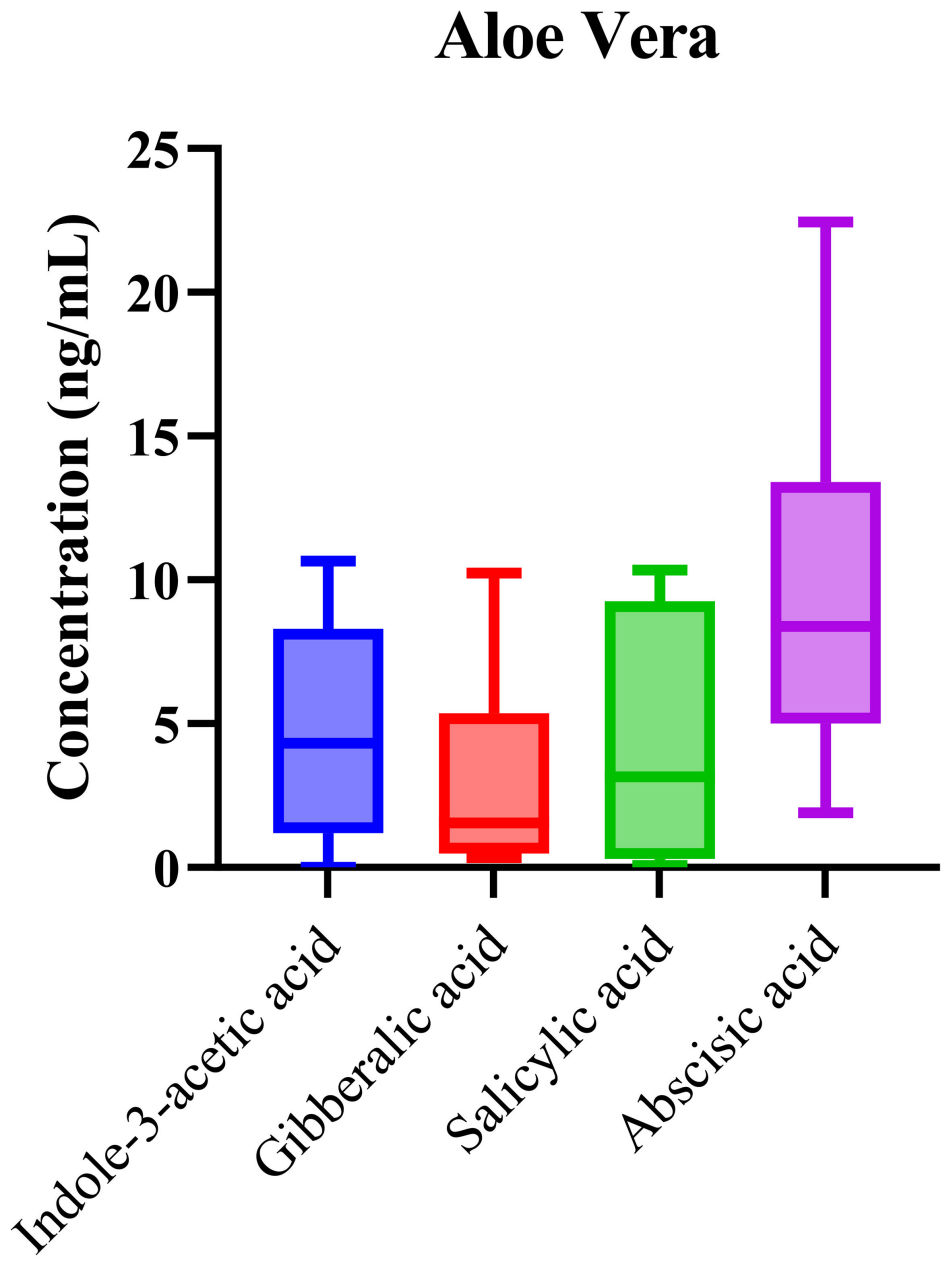
Box-and-whisker plots of phytohormonal profiles in *Aloe vera*. The data illustrate variability across analyzed samples, reflecting species-specific hormonal regulation.

Phytohormonal analysis of aloe vera samples revealed the presence of key hormones, including IAA, ABA, SA, and GA. As shown in **Figure 6**, the box-and-whisker plots demonstrate the concentration ranges of these phytohormones across the samples. While ABA and IAA exhibited higher median concentrations, the overall levels of these phytohormones were lower compared to those in the other matrices. The elevated median concentrations of abscisic acid (ABA) and indole-3-acetic acid (IAA) suggest that these hormones play significant roles in regulating aloe vera’s growth and stress responses. ABA, known for its involvement in water stress and drought tolerance, is particularly important in succulents like aloe vera, which thrive in arid environments. ABA regulates key physiological processes such as stomatal closure, which helps minimize water loss during periods of drought, making it critical for the plant’s survival in its native, water-scarce habitats. The high concentration of ABA in aloe vera aligns with the plant’s adaptation mechanisms to conserve water and thrive in desert-like conditions.

Indole-3-acetic acid (IAA), a major auxin, plays a crucial role in cell elongation, root development, and overall growth regulation. Its elevated presence in aloe vera suggests its involvement in promoting cellular growth and differentiation, particularly in the root and leaf tissues, which is essential for nutrient and water storage. The role of IAA in regulating growth is well-documented, and its higher concentration may reflect the plant’s continuous vegetative growth and recovery after water stress events. Interestingly, the relatively low concentrations of salicylic acid (SA) and gibberellic acid (GA) in aloe vera contrast with their higher concentrations in other matrices analyzed in this study. SA is generally associated with defense responses, including the regulation of systemic acquired resistance (SAR), but its low levels in aloe vera suggest that defense-related signaling might not be as active under the conditions studied, possibly due to the plant’s inherent resilience. Similarly, low GA concentrations may reflect aloe vera’s slow growth rate compared to other plants, as GA is involved in germination, stem elongation, and fruit development. However, its minimal presence could indicate that aloe vera’s growth and development processes are primarily governed by IAA and ABA, rather than GA, which may not be as critical for this species’ particular growth strategy.

Statistical analysis through one-way ANOVA revealed significant differences in phytohormone concentrations among the groups (p = 0.0058), suggesting modest variability due to environmental or developmental factors. The low variability in phytohormone concentrations across aloe vera samples suggests that aloe vera maintains a relatively stable hormonal profile despite different environmental conditions. This consistency may be due to aloe vera’s inherent physiological characteristics, such as its ability to store water and nutrients efficiently, allowing it to stabilize internal processes even when external conditions fluctuate. Unlike other plants, which may exhibit large fluctuations in phytohormone levels due to environmental stressors, aloe vera’s ability to regulate and maintain its water and nutrient balance likely contributes to its stable hormonal levels. The relatively low R² value (0.24) in the statistical model indicates a moderate fit, suggesting that genetic variability or developmental stage may play a more significant role in regulating hormonal dynamics in aloe vera than environmental factors alone. This moderate variability highlights the potential influence of internal plant factors, such as growth phase or plant health, in determining the phytohormonal profile. In comparison to other matrices, where environmental factors appeared to have a more pronounced effect on phytohormonal variation, aloe vera’s hormonal regulation may be more genetically controlled or influenced by developmental stage.

**Figure 7:**
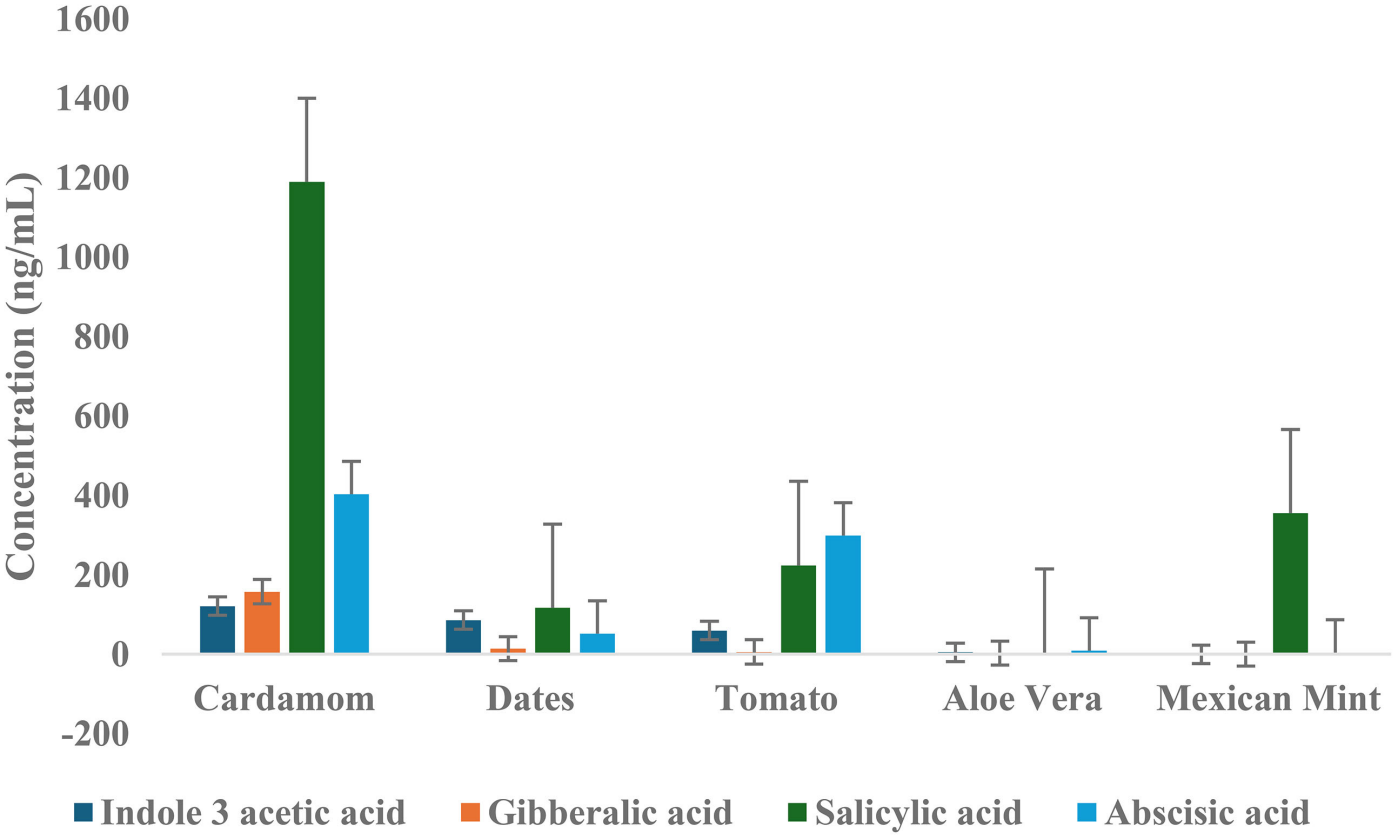
Average concentrations of key phytohormones across all five plant matrices with error bars representing standard deviation (SD) of replicate measurements, reflecting variability within each matrix.

To summarize the phytohormonal profiles, average concentrations of four key hormones, indole-3-acetic acid (IAA), gibberellic acid (GA), salicylic acid (SA), and abscisic acid (ABA), were compared across the five plant matrices analyzed (**Figure 7**). This comparative visualization highlights distinct hormone distribution patterns unique to each matrix, reflecting their specific physiological and ecological adaptations. Cardamom exhibited the highest average concentrations of SA (1189.6 ng/mL) and ABA (402.8 ng/mL), reinforcing its strong regulatory capacity in stress response and developmental processes. The date matrix showed moderate average levels of IAA (85.9 ng/mL) and SA (117.2 ng/mL), with comparatively lower concentrations of GA (14.2 ng/mL) and ABA (52.0 ng/mL). This hormone distribution aligns with dates’ physiological demands for growth regulation and stress response in arid environments. Notably, the relatively high IAA level supports its key role in fruit development and maturation processes.

Tomato samples presented elevated ABA (299.6 ng/mL) and SA (224.4 ng/mL) levels, consistent with the hormones’ established functions in fruit ripening and stress signaling. These hormone profiles corroborate the tomato’s complex developmental regulation, particularly under biotic and abiotic stress. Aloe vera exhibited the lowest concentrations for most hormones, with SA (4.5 ng/mL) and GA (3.2 ng/mL) markedly reduced relative to other matrices. The minimal hormone levels are indicative of aloe’s succulent physiology, adapted to conserve resources and maintain homeostasis in water-limited habitats. Interestingly, Mexican mint demonstrated a uniquely high average SA concentration (355.3 ng/mL) despite very low levels of IAA, GA, and ABA. This pattern reflects the plant’s specialized, defense-oriented metabolism, consistent with its medicinal properties and adaptation to environments rich in pathogens. This comparative analysis (**Figure 7**) elucidates the diverse hormonal landscapes across plant matrices, highlighting how phytohormone abundance correlates with species-specific growth strategies and environmental interactions. These findings lay the groundwork for interpreting the functional implications of hormonal variation in plant physiology and agricultural practices.

### Correlation analysis of phytohormone profiles across plant matrices

**Figure 8:**
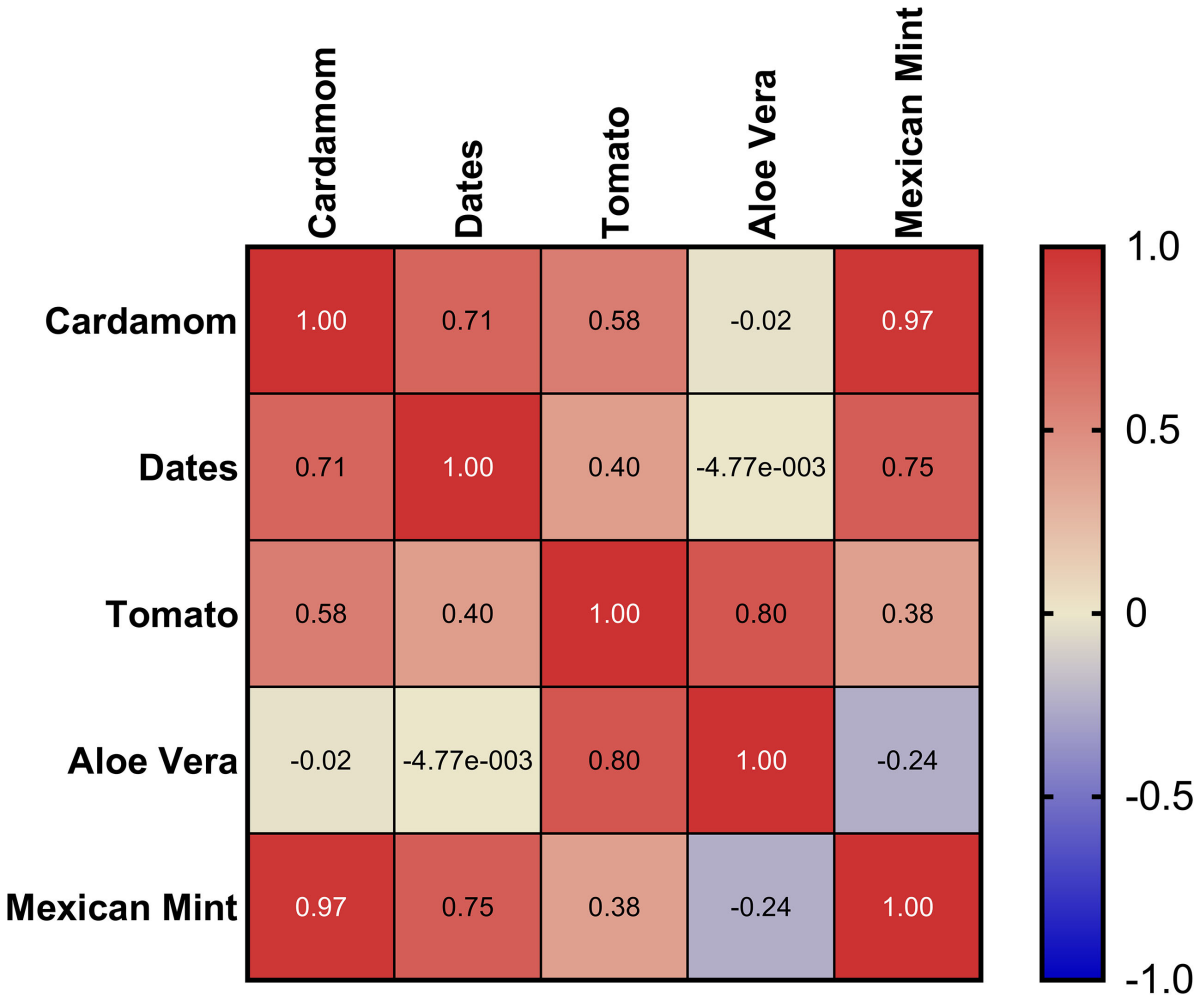
Pearson correlation heatmap showing relationships among phytohormone profiles across five plant matrices. Values range from +1, indicating a perfect positive correlation, to -1, indicating a perfect negative correlation, with 0 representing no correlation.

To further explore the relationships among the phytohormonal profiles across the different plant matrices, a Pearson correlation analysis was conducted (**Figure 8**). The heatmap depicts pairwise Pearson correlation coefficients (r) calculated from average phytohormone concentrations measured in all matrices. High positive correlations (values close to +1) suggest similar hormone distribution patterns between matrices, whereas values near -1 indicate inverse relationships. The resulting correlation heatmap reveals strong positive correlations between cardamom and Mexican mint (r = 0.97), as well as between cardamom and dates (r = 0.71), indicating similar hormone distribution patterns between these matrices. Moderate correlations were observed between tomato and aloe vera (r = 0.80), suggesting some shared hormonal regulation despite their distinct physiological traits.

Notably, aloe vera showed negligible or slightly negative correlations with cardamom (r = -0.02) and dates (r ≈ 0), reflecting its unique hormonal profile, consistent with its succulent nature and adaptation to arid conditions. This divergence emphasizes aloe vera’s distinct regulatory mechanisms compared to the other matrices. This analysis highlights the heterogeneity of phytohormonal profiles across the studied matrices, suggesting that species-specific factors and environmental adaptations heavily influence hormone regulation. The high correlations between cardamom, Mexican mint, and dates may reflect comparable stress responses or developmental pathways, while aloe vera’s distinctive profile underscores its specialized survival strategy in water-limited environments.

### Biological and agricultural implications

The findings of this study underscore the complex interplay between phytohormones, environmental factors, and plant growth strategies. In all five matrices, the regulation of key hormones reflects the plants’ efforts to adapt to and survive in their respective environments. Salicylic acid (SA), in particular, was found to be a crucial regulator of stress responses, mediating plant adaptation to harsh conditions such as drought, temperature extremes, and pathogen attacks.

Understanding how these phytohormones regulate growth, stress responses, and fruit ripening can help optimize agricultural practices, particularly in the cultivation of economically significant plants such as dates and tomatoes, which are highly sensitive to environmental stress. The variability in phytohormone levels across matrices suggests that regional cultivation practices and environmental conditions should be taken into account when developing strategies to improve crop quality and yield. Additionally, this study offers critical insights into the medicinal properties of plants such as Mexican mint and aloe vera, where SA and ABA play crucial roles in regulating the plant’s pharmacological activity. Future research into hormonal regulation in these plants could lead to more effective methods for optimizing their therapeutic properties.

## Conclusion

This study presents a comprehensive analysis of phytohormonal profiles across five distinct plant matrices: cardamom, dates, tomato, Mexican mint, and aloe vera, using a unified LC-MS/MS analytical platform with consistent chromatographic and mass spectrometric conditions combined with tailored, matrix-specific extraction procedures. The findings reveal that each matrix exhibits a unique phytohormonal signature shaped by environmental conditions, genetic factors, and its inherent physiological requirements. By investigating the key phytohormones such as indole-3-acetic acid (IAA), abscisic acid (ABA), salicylic acid (SA), and gibberellic acid (GA), this study provides valuable insights into how plants regulate growth, stress responses, and reproductive processes. The comparative analysis of phytohormonal profiles across diverse plant matrices reveals both commonalities and divergences in hormonal regulation. While stress-related hormones, such as SA and ABA, play central roles across all matrices, their concentrations and significance vary significantly depending on the plant species and environmental conditions. These findings underscore the need for future research to investigate the interplay between genetic, environmental, and physiological factors in shaping phytohormonal regulation. The insights gained from this study provide a valuable foundation for advancing our understanding of plant stress responses and growth regulation, with implications for improving both crop production and medicinal plant cultivation.

### Future directions

Future research should aim to expand on the findings of this study by investigating the role of genetic diversity in phytohormonal regulation within different species, which could provide valuable insights into how genetic factors contribute to variation in phytohormonal profiles. Genetic mapping of key phytohormone-related genes may help identify molecular markers that can be used to enhance stress tolerance and growth regulation in crops, facilitating precision breeding for resilient plant varieties. Additionally, while this study has explored the impact of environmental conditions on phytohormonal profiles, further research is needed to understand how specific environmental stressors, such as heat stress and salinity, affect phytohormone regulation in different species. Understanding these interactions can help in climate-resilient crop breeding strategies aimed at mitigating the effects of climate change on crop yield and quality.

Moreover, investigating how hormones such as salicylic acid (SA) and abscisic acid (ABA) interact to modulate drought tolerance and other stress responses could open new avenues for enhancing stress resilience in crops. Finally, developing high-throughput techniques for analyzing phytohormones across a broader range of species will be crucial for conducting large-scale studies on plant physiology and hormone regulation. Such techniques would be particularly valuable for agricultural systems globally, especially in the context of climate change, as they would facilitate the rapid assessment of phytohormonal profiles and help optimize crop productivity. Exploring these areas will not only deepen our understanding of plant physiology but also provide practical solutions to enhance crop yield, quality, and medicinal applications, contributing to the sustainability of global agriculture.

## Data Availability

The original contributions presented in the study are included in the article/**Supplementary Material**. Further inquiries can be directed to the corresponding authors.
