# Supplementary material for: Comparative LC-MS/MS-based profiling of phytohormones: a unified analytical approach across diverse plant matrices
Source: Front Plant Sci. 2025 Sep 19;16:1670979. doi: 10.3389/fpls.2025.1670979 (PMC12491226; doi:10.3389/fpls.2025.1670979)
Supplement: Supplementary file 1 [file Supplementaryfile1.docx]

**Supplementary Information**

**Comparative LC-MS/MS-Based Profiling of Phytohormones: A Unified Analytical Approach Across Diverse Plant Matrices**

Muhammad K. Hakeem ^1^, Tamilarasan Rajendaran ^2^, Esam Eldin Saeed ^2^, Ajay K. Mishra ^2^, Khaled M. Hazzouri ^2^, Iltaf Shah ^1,2, *^, Khaled M. A. Amiri ^2,3, *^

^1^ Department of Chemistry, College of Science, United Arab Emirates University, Al Ain, 15551, United Arab Emirates

^2^ Khalifa Center for Genetic Engineering and Biotechnology, United Arab Emirates University, Al Ain, United Arab Emirates

^3^ Department of Biology, College of Science, United Arab Emirates University (UAEU), Al Ain P.O. Box 15551.United Arab Emirates

Muhammad Kamran Hakeem: 700039966@uaeu.ac.ae

Tamilarasan Rajendaran: tamil.r@uaeu.ac.ae

Esam Eldin Saeed: esameldin_saeed@uaeu.ac.ae

Ajay Kumar Mishra: ajaymishra24@uaeu.ac.ae

Khaled M. Hazzouri: [khaled_hazzouri@uaeu.ac](mailto:khaled_hazzouri@uaeu.ac).

Iltaf Shah: [altafshah@uaeu.ac.ae](mailto:altafshah@uaeu.ac.ae)

Khaled M. A. Amiri: [k.amiri@uaeu.ac.ae](mailto:k.amiri@uaeu.ac.ae)

* Correspondence: altafshah@uaeu.ac.ae ; k.amiri@uaeu.ac.ae

**Supplementary Table S1: Comprehensive Sample Preparation and Extraction Protocols for Each Matrix**

| **Matrix** | **Extraction Solvent System** |
| --- | --- |
| **Cardamom** | 1% Acetic acid in ethanol (5 mL) + 1 mL Milli-Q H_2_O |
| **Dates** | Acetic acid (1 mL) + 2% HCl in ethanol (4 mL) + 80:20 MeOH: 0.1% FA in H_2_O |
| **Tomato** | 1% Acetic acid in Acetonitrile (10 mL) + 35:65 0.1% FA in H_2_O: MeOH |
| **Mexican Mint** | 1% Formic Acid in Methanol (2.5 mL) + 500 µL Milli-Q H_2_O |
| **Aloe Vera** | 1% Acetic Acid in Acetonitrile (5.0 mL) |

SA-D4: Salicylic Acid-D4; FA: Formic Acid; MeOH: Methanol

**Supplementary Table S2: LC-MS/MS Analysis Parameters**

| **Compound** | **Q1 (m/z)** | **Q3 (m/z)** | **Dwell Time (ms)** | **Q1 Pre-Bias (V)** | **CE (V)** | **Q3 Pre-Bias (V)** | **ESI Polarity** |
| --- | --- | --- | --- | --- | --- | --- | --- |
| **Indole-3-Acetic Acid (IAA)** | 176 | 130.1 | 100 | -17 | -15 | -22 | + |
|  | 176 | 77.1 | 100 | -16 | -42 | -15 | + |
|  | 176 | 103.05 | 100 | -16 | -29 | -23 | + |
| **Isopentenyl Adenine (IPA)** | 204.15 | 136 | 100 | -20 | -15 | -20 | + |
|  | 204.15 | 148 | 100 | -10 | -13 | -14 | + |
|  | 204.15 | 119 | 100 | -10 | -31 | -11 | + |
| **Naphthalene Acetic Acid (NAA)** | 184.9 | 117.05 | 100 | 22 | 11 | 26 | - |
|  | 184.9 | 100.05 | 100 | 20 | 34 | 38 | - |
|  | 184.9 | 141.1 | 100 | 22 | 10 | 20 | - |
| **Indole-3-Butyric Acid (I3BA)** | 202.05 | 134.15 | 100 | 22 | 16 | 20 | - |
|  | 202.05 | 133.15 | 100 | 22 | 22 | 20 | - |
|  | 202.05 | 132.2 | 100 | 22 | 30 | 28 | - |
| **6-Benzyl Aminopurine (6BAP)** | 224.05 | 133.15 | 100 | 24 | 23 | 26 | - |
|  | 224.05 | 132.15 | 100 | 24 | 32 | 26 | - |
|  | 224.05 | 188 | 100 | 26 | 13 | 22 | - |
| **Gibberellic Acid (GA)** | 345.25 | 143.2 | 100 | 17 | 29 | 13 | - |
|  | 345.25 | 239.2 | 100 | 17 | 16 | 30 | - |
|  | 345.25 | 221.35 | 100 | 17 | 25 | 22 | - |
| **Salicylic Acid (SA)** | 137.2 | 93.15 | 100 | 13 | 21 | 11 | - |
|  | 137.2 | 65.2 | 100 | 14 | 28 | 15 | - |
|  | 137.2 | 100 | 100 | 15 | 27 | 26 | - |
| **Abscisic Acid (ABA)** | 263.1 | 153.25 | 100 | 28 | 12 | 20 | - |
|  | 263.1 | 219.25 | 100 | 28 | 14 | 24 | - |
|  | 263.1 | 204.3 | 100 | 28 | 20 | 26 | - |
| **Salicylic Acid D4 (IS)** | 141.1 | 97.1 | 100 | 14 | 21 | 12 | - |
|  | 141.1 | 59.1 | 100 | 14 | 12 | 14 | - |
|  | 141.1 | 69.15 | 100 | 14 | 30 | 8 | - |

**Supplementary Table S3: Validation Results for Cardamom Matrix**

| **Compound** | **Linearity Range (ng/mL)** | **R^2^** | **LOD (ng/mL)** | **LOQ (ng/mL)** | **Concentration (ng/mL)** | **RSD %(< 20 %)** | **Recovery % (80-120)** |
| --- | --- | --- | --- | --- | --- | --- | --- |
| **Indole 3 acetic acid** | 10 - 125 | 0.999 | 3.705 | 11.220 | 9.840 | 3.255 | 98.402 |
|  |  |  |  |  | 21.219 | 8.384 | 106.094 |
|  |  |  |  |  | 40.131 | 4.768 | 80.261 |
| **Gibberellic acid** | 10 - 125 | 0.993 | 2.374 | 7.196 | 9.590 | 9.190 | 95.904 |
|  |  |  |  |  | 22.512 | 7.296 | 112.559 |
|  |  |  |  |  | 54.205 | 9.826 | 108.409 |
| **Salicylic acid** | 40 - 500 | 0.994 | 2.155 | 6.531 | 9.522 | 2.398 | 95.216 |
|  |  |  |  |  | 19.724 | 2.615 | 98.619 |
|  |  |  |  |  | 58.169 | 6.359 | 116.337 |
| **Abscisic acid** | 40 - 500 | 0.997 | 3.705 | 11.220 | 10.746 | 3.893 | 107.458 |
|  |  |  |  |  | 14.502 | 1.896 | 82.508 |
|  |  |  |  |  | 41.290 | 4.531 | 82.579 |

**Supplementary Table S4: Validation Results for Dates Matrix**

| **Compound** | **Linearity Range (ng/mL)** | **R^2^** | **LOD (ng/mL)** | **LOQ (ng/mL)** | **Concentration (ng/mL)** | **RSD %(< 20 %)** | **Recovery % (80-120)** |
| --- | --- | --- | --- | --- | --- | --- | --- |
| **Indole 3 acetic acid** | 5 to 100 | 0.998 | 2.374 | 7.196 | 10.457 | 2.502 | 104.567 |
|  |  |  |  |  | 19.491 | 6.567 | 97.453 |
|  |  |  |  |  | 43.530 | 3.720 | 87.059 |
| **Gibberellic acid** | 5 to 100 | 0.991 | 2.155 | 6.531 | 9.983 | 3.178 | 99.827 |
|  |  |  |  |  | 19.187 | 7.131 | 95.933 |
|  |  |  |  |  | 58.843 | 7.928 | 117.685 |
| **Salicylic acid** | 5 to 100 | 0.998 | 2.731 | 8.278 | 10.967 | 1.567 | 109.667 |
|  |  |  |  |  | 20.437 | 1.064 | 102.183 |
|  |  |  |  |  | 47.844 | 0.162 | 95.687 |
| **Abscisic acid** | 5 to 100 | 0.991 | 3.682 | 11.150 | 10.700 | 4.932 | 106.998 |
|  |  |  |  |  | 20.284 | 1.418 | 101.422 |
|  |  |  |  |  | 48.147 | 9.361 | 96.294 |

**Supplementary Table S5: Validation Results for Tomato Matrix**

| **Compound** | **Linearity Range (ng/mL)** | **R^2^** | **LOD (ng/mL)** | **LOQ (ng/mL)** | **Concentration (ng/mL)** | **RSD %)(< 20 %)** | **Recovery % (80-120)** |
| --- | --- | --- | --- | --- | --- | --- | --- |
| **Indole 3 acetic acid** | 10 to 225 | 0.997 | 2.631 | 7.974 | 10.256 | 8.230 | 102.560 |
|  |  |  |  |  | 21.486 | 7.960 | 107.430 |
|  |  |  |  |  | 46.780 | 2.320 | 93.560 |
| **Gibberellic acid** | 5 to 100 | 0.990 | 4.044 | 12.250 | 11.047 | 20.020 | 110.470 |
|  |  |  |  |  | 21.620 | 6.570 | 108.100 |
|  |  |  |  |  | 48.215 | 9.480 | 96.430 |
| **Salicylic acid** | 5 to 250 | 0.995 | 2.590 | 7.851 | 8.873 | 11.870 | 88.730 |
|  |  |  |  |  | 18.820 | 9.510 | 94.100 |
|  |  |  |  |  | 49.870 | 0.760 | 99.740 |
| **Abscisic acid** | 5 to 100 | 0.9958 | 2.656 | 8.049 | 9.008 | 3.840 | 90.080 |
|  |  |  |  |  | 23.054 | 6.500 | 115.270 |
|  |  |  |  |  | 47.600 | 3.280 | 95.200 |
| **6-Benzyl aminopurine** | 5 to 100 | 0.9895 | 3.139 | 9.513 | 10.615 | 15.550 | 106.150 |
|  |  |  |  |  | 20.422 | 10.570 | 102.110 |
|  |  |  |  |  | 49.250 | 3.730 | 98.500 |
| **Naphthalene acetic acid** | 5 to 100 | 0.9962 | 2.922 | 8.855 | 9.372 | 4.150 | 93.720 |
|  |  |  |  |  | 20.340 | 2.160 | 101.700 |
|  |  |  |  |  | 51.675 | 2.060 | 103.350 |
| **Isopentenyl adenine** | 0.2 to 35 | 0.993 | 0.010 | 0.040 | 0.160 | 2.110 | 95.650 |
|  |  |  |  |  | 0.320 | 4.380 | 116.250 |
|  |  |  |  |  | 0.800 | 3.630 | 96.680 |

**Supplementary Table S6: Validation Results for Mexican Mint Matrix**

| **Compound** | **Linearity Range (ng/mL)** | **R^2^** | **LOD (ng/mL)** | **LOQ (ng/mL)** | **Concentration (ng/mL)** | **RSD % (< 20 %)** | **Recovery % (80-120)** |
| --- | --- | --- | --- | --- | --- | --- | --- |
| **Indole 3 acetic acid** | 1 to 200 | 0.999 | 2.149 | 6.514 | 9.643 | 9.48 | 96.43 |
|  |  |  |  |  | 20.512 | 8.23 | 102.56 |
|  |  |  |  |  | 53.715 | 7.96 | 107.43 |
| **Gibberellic acid** | 1 to 100 | 0.999 | 2.626 | 7.959 | 9.974 | 0.76 | 99.74 |
|  |  |  |  |  | 22.094 | 20.02 | 110.47 |
|  |  |  |  |  | 54.05 | 6.57 | 108.1 |
| **Salicylic acid** | 1 to 200 | 0.999 | 2.639 | 7.999 | 9.52 | 3.28 | 95.2 |
|  |  |  |  |  | 17.746 | 11.87 | 88.73 |
|  |  |  |  |  | 47.05 | 9.51 | 94.1 |
| **Abscisic acid** | 1 to 100 | 0.999 | 3.429 | 10.39 | 9.008 | 3.84 | 90.08 |
|  |  |  |  |  | 19.136 | 9.51 | 95.678 |
|  |  |  |  |  | 57.635 | 6.5 | 115.27 |
| **6-Benzyl aminopurine** | 1 to 100 | 0.998 | 4.003 | 12.13 | 10.615 | 15.55 | 106.15 |
|  |  |  |  |  | 20.422 | 10.57 | 102.11 |
|  |  |  |  |  | 49.25 | 3.73 | 98.5 |
| **Indole-3-butyric acid** | 5 to 100 | 0.999 | 2.174 | 6.59 | 8.617 | 7.52 | 86.17 |
|  |  |  |  |  | 18.658 | 13.72 | 93.29 |
|  |  |  |  |  | 55.54 | 9.13 | 111.08 |
| **Naphthalene acetic acid** | 100 to 2500 | 0.996 | 2.475 | 7.502 | 131.68 | 2.32 | 93.56 |
|  |  |  |  |  | 263.36 | 4.15 | 93.72 |
|  |  |  |  |  | 1316.8 | 2.16 | 101.7 |
| **Isopentenyl adenine** | 0.2 to 35 | 0.999 | 3.057 | 9.265 | 0.16 | 3.796 | 96.505 |
|  |  |  |  |  | 3.205 | 3.941 | 94.758 |
|  |  |  |  |  | 5.213 | 1.847 | 93.763 |

**Supplementary Table S7: Validation Results for Aloe Vera Matrix**

| **Compound** | **Linearity Range (ng/mL)** | **R^2^** | **LOD (ng/mL)** | **LOQ (ng/mL)** | **Concentration (ng/mL)** | **RSD % (< 20 %)** | **Recovery % (80-120)** |
| --- | --- | --- | --- | --- | --- | --- | --- |
| **Indole 3 acetic acid** | 1 to 30 | 0.989 | 0.270 | 0.830 | 2.070 | 3.710 | 89.960 |
|  |  |  |  |  | 4.140 | 2.020 | 107.120 |
|  |  |  |  |  | 10.350 | 3.440 | 111.340 |
| **Gibberellic acid** | 1 to 30 | 0.995 | 0.330 | 1.010 | 2.020 | 4.920 | 90.530 |
|  |  |  |  |  | 4.030 | 9.180 | 90.830 |
|  |  |  |  |  | 10.080 | 3.140 | 95.640 |
| **Salicylic acid** | 1 to 30 | 0.993 | 0.540 | 1.620 | 1.060 | 14.680 | 90.430 |
|  |  |  |  |  | 2.120 | 9.510 | 89.450 |
|  |  |  |  |  | 5.300 | 4.740 | 86.130 |
| **Abscisic acid** | 1 to 30 | 0.996 | 0.400 | 1.200 | 0.990 | 10.080 | 105.970 |
|  |  |  |  |  | 1.990 | 4.740 | 103.820 |
|  |  |  |  |  | 4.990 | 2.340 | 100.150 |
| **6-Benzyl aminopurine** | 1 to 25 | 0.991 | 0.510 | 1.540 | 2.560 | 7.540 | 84.590 |
|  |  |  |  |  | 5.120 | 12.200 | 97.220 |
|  |  |  |  |  | 12.810 | 1.860 | 100.280 |
| **Naphthalene acetic acid** | 125 to 2500 | 0.994 | 36.400 | 110.300 | 126.620 | 4.550 | 101.670 |
|  |  |  |  |  | 253.230 | 1.830 | 100.530 |
|  |  |  |  |  | 633.080 | 2.270 | 101.660 |
| **Isopentenyl adenine** | 0.2 to 35 | 0.993 | 0.010 | 0.040 | 0.160 | 2.110 | 95.650 |
|  |  |  |  |  | 0.320 | 4.380 | 116.250 |
|  |  |  |  |  | 0.800 | 3.630 | 96.680 |

*RSD: Relative Standard Deviation; LOD: limits of detection; LOQ: limits of quantification*
